# Aspects of General Practice Studied by University of Bristol Students

**Published:** 1983-07

**Authors:** Robin Philipp, Anthony Hughes

**Affiliations:** Senior Lecturer in Medical Statistics, University of Bristol


### Aspects of General Practice Studied by University of Bristol Students

**Robin Philipp M.F.C.M.I. A.F.O.M. M.Sc. D.C.H.**
Lecturer in Community Medicine
**Anthony Hughes M.Sc. M.Phil.**
Senior Lecturer in Medical Statistics, University of Bristol.

During the final year of the undergraduate curriculum in the University of Bristol, all final year medical students are attached to the Department of Community Health for a full-time, 9-week, 'Medicine in the Community' course. The teaching on this course includes epidemiology and medical statistics, the principles of medical care systems, occupational and environmental health, and General Practice. Details of the course and its objectives have been previously described (Lennard, 1975; Macara, 1973). Although the University of Bristol does not yet have an academic Department of General Practice (Whitfield, 1982), and it is not certain what arrangements are best suited within universities for the provision of undergraduate experience of General Practice (Marinker, 1983), the objectives of the University of Bristol General Practice attachments are similar to those reported for academic departments of General Practice (Harris 1983).

Evaluations of the 9-week 'Medicine in the Community' course have been undertaken since 1975 to measure whether the course objectives are met. These evaluations have shown that students consider that the General Practice attachments are the most valuable and interesting aspect of the course (Macara and Philipp, 1976; Philipp, 1976). Interest in a subsequent career in General Practice also seems to be encouraged; between 1974 and 1979, the proportion of newly qualified doctors from the University of Bristol who expressed a career preference for General Practice ranked from eighth highest to first place amongst 29 medical schools in the U.K. (Parkhouse, Palmer and Hambleton, 1979); (Faragher, Parkhouse and Parkhouse, 1980); (Parkhouse, Campbell and Phillips, 1981). For doctors qualifying in 1980, however, this ranking placed Bristol graduates in 18th place. (Parkhouse, Campbell, Hambleton and Phillips, 1983). A further measure of student interest in General Practice is their success in the Royal College of General Practitioners Annual Undergraduate Essay Prize. Since the competition was introduced in 1973, it has been won by a Bristol student twice, and second and third places gained on two and four occasions respectively. The College has also advised the University of Bristol that this competition attracts the highest number of entries from Bristol students.

The General Practice attachments were last evaluated in 1976. A further evaluation was undertaken in the 1982–83 academic year and its findings are reported here.

### METHOD

Between November 1982 and April 1983, 83 students attended the 'Medicine in the Community' course in the Department of Community Health, University of Bristol. As part of this course, there are two attachments to Teachers in General Practice. The first of these consists of 14 sessional attachments to a General Practitioner in Bristol that take place over a 3-week period. The other placement which follows the Bristol attachment, is a 2-week, usually residential, attachment to a General Practitioner elsewhere in the region. The students were asked to complete a questionnaire at the end of the second attachment. This questionnaire listed 26 aspects of General Practice. They were asked to indicate whether or not they had studied each aspect during the Bristol, and the Regional attachment. These 26 aspects of the attachments compromised five groupings:

(1) *Clinical aspects;* clinical problem solving and management, antenatal care, care of the sick child, effects of occupation on health, management of psychiatric illness, care of the elderly, care of the dying and bereaved, immunisation and vaccination practice, screening for disease, preventive medical advice.

(2) *Case discussion and visiting;* case discussions between medical practitioners, case discussions with non-medical professional staff, home visiting of patients, hospital visiting of patients.

(3) *Organisation and research;* practice organisation, practice audit, research work in general practice, the G.P. and the law, medical ethics.

(4) *Paramedical contact;* the work of a health visitor, the work of a practice or clinic nurse, the work of a district nurse, work of a social worker.

**Table 1**

**Differences between aspects of General Practice studied by 73 students in their Bristol and Regional attachments**

| | *Bristol attachment* | | *Regional attachment* | | |
|---|---|---|---|---|---|
| *Aspect of General Practice* | *Studied* | *Not studied* | *Studied* | *Not studied* | *P value* |
| Care of the dying and bereaved | 48 | 25 | 62 | 11 | * |
| Immunisation and vaccination practice | 46 | 27 | 61 | 12 | ** |
| Hospital visiting of patients | 11 | 62 | 35 | 38 | *** |
| Practice organisation | 62 | 11 | 70 | 3 | * |
| Practice audit | 25 | 48 | 40 | 33 | * |
| Research work | 16 | 57 | 31 | 42 | * |
| The G.P. and the law | 26 | 47 | 40 | 33 | * |
| The work of a practice/clinic nurse | 42 | 31 | 58 | 15 | ** |
| The work of a social worker | 53 | 20 | 69 | 4 | *** |

$^{*}p<0.05$; $^{**}p<0.01$; $^{***}p<0.001$.

(5) *Lifestyle;* interpersonal behaviour between doctors and patients, interpersonal behaviour between patient and family or friends, lifestyle of a G.P.

Students were asked to interpret 'studied/not studied', as any contact with that aspect of General Practice.

### RESULTS

Of the 83 students, 73 (88%) completed and returned the questionnaire. For nine of the 26 aspects of General Practice, there were statistically significant differences between the number of students who did or did not study them during the Bristol attachments when compared with whether or not they were studied during the regional attachments. These aspects of the General Practice attachments, and the number of students who did or did not study them in Bristol and the Region, are shown on Table 1.

It was considered particularly worthwhile to look for any aspects of General Practice that students indicated had not been studied in either the Bristol or Regional attachments. Of the 26 aspects that were examined, 23 had not been studied by at least one student. For seven of these 26 aspects, more than 20% of the students did not study them. These seven aspects of General Practice, together with the number of students who did not study each of them, are shown in Table 2.

**Table 2**

**Aspects of General Practice that 20% (15/73) or more students did not study in either the Bristol or Regional G. P. attachment**

| *Aspect of General Practice considered by 73 students* | *Number of students who did not study this aspect* |
|---|---|
| Effects of occupation on health | 20 |
| Case discussions between medical practitioners | 17 |
| Hospital visiting of patients | 34 |
| Practice audit | 28 |
| Research work in general practice | 35 |
| The G.P. and the law | 30 |
| The work of a social worker | 28 |

### DISCUSSION

The Bristol and Regional attachments in General Practice are, wherever possible, chosen to provide different experiences for an individual student. The arrangements for each attachment are also different; as noted earlier, the Bristol attachments are undertaken on a sessional basis and these are not consecutive, whereas the Regional attachment consists of a 2-week, usually residential, placement. Consistently more students studied each aspect of General Practice during their Regional attachment, as compared with the Bristol attachment. There may be several reasons for this difference. For example, students may not avail themselves of the proffered opportunity, an individual aspect of General Practice may not be available in some practices, or suitable opportunities to study an individual aspect may not become available during the limited contact time between students and their Teachers in General Practice. Furthermore, the Bristol attachment precedes the Regional placement. It is therefore likely that students identify aspects of General Practice in Bristol that they would like to study but are unable to do so, for example because of limitations of time or the nature of work undertaken during the available sessions. An opportunity to study aspects may arise during the Regional attachment.

It is perhaps of concern that at least one fifth of the students did not study seven of the 26 aspects during either of the two attachments. Whereas it is hoped that missed opportunities, or those that are not available during one attachment, can be identified during the other placement, this does not always occur. The findings in Table 2 show that a substantial proportion of students have not seen some aspects of General Practice by the end of both their attachments. It is therefore possible that closer collaboration between the two General Practitioners to whom any one student is attached, could ensure that no major gaps in experience remain at the end of these attachments. In spite of these deficiences the students, at the end-of-course evaluation, expressed their satisfaction with the present arrangements. Although they acknowledged that individual General Practitioners make different arrangements for students, they were adamant that further structuring of the attachments should not be undertaken. Nevertheless, the results of this study suggest that for some students the quality of undergraduate experience in General Practice could be improved.

### REFERENCES

1. FARAGHER, E. B., PARKHOUSE, J. and PARKHOUSE, H. F., (1980) Career preferences of doctors qualifying in the U.K. in 1978. *Health Trends* **12**, 101–102.
2. HARRIS, C. (1983) The G.P. and the medical student: the background, *British Medical Journal* **1**, 111.
3. LENNARD, M. B., (1975) Medicine in the community—an undergraduate course. *The New Zealand Family Physician* **2**, 21.
4. MACARA, A. W. (1973) The teaching of Community Medicine to medical undergraduates. *Community Health* **4**, 278.
5. MACARA, A. W. AND PHILIPP, R. (1976) Student audit: uses and limitations of an evaluation questionnaire. *Medical Education* **10**, 137.
6. MARINKER, M. (1983) Should general practice be represented in the University Medical School? *British Medical Journal* **1**, 855–859.
7. PARKHOUSE, J., CAMPBELL, M. G., HAMBLETON, B. A. and PHILLIPS, P. R. (1983) Career preferences of doctors qualifying in the United Kingdom in 1980. *Health Trends* **15**, 12–14.
8. PARKHOUSE, J., CAMPBELL, M. G. and PHILLIPS, P. R. (1981) Career preferences of doctors qualifying in the United Kingdom in 1979. *Health Trends* **13**, 103–105.
9. PARKHOUSE, J., PALMER, M. K. and HAMBLETON, B. A. (1979) Career preferences of doctors qualifying in the United Kingdom in 1977. *Health Trends* **11**, 35–37.
10. PHILIPP, R. (1976) Attitude modification with education. *Medical Education* **10**, 524–527.
11. WHITFIELD, M. (1982) A Department of General Practice at Bristol? *The Royal College of General Practitioners Severn Faculty Newsletter* No. 4, 5–6.

